# Update on Bacterial Blight of Rice: Fourth International Conference on Bacterial Blight

**DOI:** 10.1186/s12284-014-0012-7

**Published:** 2014-07-26

**Authors:** Raman M Sundaram, Subhadeep Chatterjee, Ricardo Oliva, Gouri Sankar Laha, Casiana Vera Cruz, Jan E Leach, Ramesh V Sonti

**Affiliations:** Directorate of Rice Research, Rajendranagar, Hyderabad, 500030 India; Centre for DNA Fingerprinting & Diagnostics, 4-1-714 Mozamjahi Rd, Hyderabad, 500 001 India; International Rice Research Institute, Manila, Philippines; Colorado State University, Ft. Collins, 80523-1177 CO USA; CSIR-Centre for Cellular and Molecular Biology, Uppal Road, Hyderabad, 500017 India

Bacterial blight (BB) of rice caused by *Xanthomonas oryzae* pv. *oryzae* (*Xoo*) is one of the most serious production constraints of rice worldwide. Besides the economic importance of the disease, the rice-*Xoo* system serves as an excellent model for studying host-pathogen interactions because the genomes of both interacting partners are sequenced and there is considerable genetic variation in the host and pathogen. The Fourth International Conference on Bacterial Blight (4th ICBB) of Rice was held in Hyderabad, India on 2–4 December 2013, and was jointly organized by the Centre for Cellular and Molecular Biology (CCMB), Directorate of Rice Research (DRR) and the Society for the Advancement of Rice Research. The 200 attendees represented 60 different institutions comprising universities, research institutes, and seed companies. The three-day conference provided an ideal atmosphere to discuss the latest developments in epidemiology, bacteria-rice interactions, and molecular breeding aspects of this important disease.

The technical sessions began with a keynote presentation by Jan Leach from Colorado State University who summarized the research progress in bacterial blight (BB) during the last 25 years. Highlighting current challenges, she stressed the need for more accurate diagnostic tools to support surveillance strategies, standardization of phenotyping protocols among labs, and the consolidation of a centralized stock centre for bacterial strains. Leach also presented transcriptional data suggesting that exposure to high temperatures reduces effectiveness of most rice BB resistance genes, but that one resistance gene, called *Xa7*, remains effective under high temperatures. Casiana Vera Cruz, from the International Rice Research Institute (IRRI), provided evidence that in addition to high temperatures, drought also differently affects efficacy of *Xa4* and *Xa7.* Combinations of *Xa4* and *Xa7* in gene pyramids provided even greater resistance at both high temperatures and in drought conditions.

## Host-plant resistance and translational research

Resistance breeding is the only viable strategy for managing BB and the topic of host-plant resistance was covered widely by many speakers. K.K. Jena from IRRI talked about pyramiding multiple resistance genes in elite Japonica rice cultivars. Gene-pyramid lines containing *Xa4, xa5* and *Xa21* showed broad-spectrum resistance with no yield penalty. Furthermore, his group has identified a new, major resistance gene named *Xa39(t)* from an accession of *O. rufipogon* that could be deployed in gene-pyramiding programs.

Dr. Kuldeep Singh from Punjab Agricultural University talked about BB resistance genes from wild relatives of *Oryza* and their introgression into elite cultivars of *O. sativa*. A new gene, *Xa38* was introgressed from an accession of *O. nivara* and pyramided along with *xa13* and *Xa21* into the elite rice variety, PAU 201, through marker-assisted breeding (MAB). Two resistance genes identified from an accession of *O. rufipogon* are being fine mapped. At least two recessive resistance genes were transferred from *O. glaberrima* to *O. sativa.* T. Mohapatra from Central Rice Research Institute in India talked about gene-pyramiding four major resistance genes, viz., *Xa21, xa13, xa5* and *Xa4* in two elite rice varieties, Lalat and Tapaswini using MAB. Similarly, three major resistance genes- *Xa21, xa13* and *xa5* have been transferred to two popular varieties- Swarna and IR64.

A.K. Singh from the Indian Agricultural Research Institute discussed the use of MAB to introduce the *Xa21* and *xa13* resistance genes into the genetic background of an Indian Basmati variety, Pusa Basmati 1. To diversify the resistance genes deployed, two wild rice derived genes, *Xa33* and *Xa38* are also being deployed in addition to *Xa21* and *xa13* in BB resistant Basmati varieties. R.M. Sundaram from DRR talked about collaborative work with CCMB in the introduction of BB resistance into Samba Mahsuri (SM), an elite Indian mega-variety possessing high yield, fine-grain type, excellent cooking and eating qualities. The *Xa21, xa13* and *xa5* genes were introgressed into the genetic background of SM through MAB. The effort culminated in the development of an improved variety which possessed yield, morphological traits and grain and cooking quality identical to SM as well as a high level of resistance against BB disease.

BB disease has spread to many non-traditional areas in India, in addition to recurring incidence in the traditional areas under irrigated and rainfed shallow lowlands (G.S. Laha, Directorate of Rice Research). Pathotyping analysis of *Xoo* isolates collected from diverse geographical locations across India has revealed at least 22 pathotypes. A new gene, *Xa33* was identified from an accession of *O. nivara* and introgressed into the genetic background of Samba Mahsuri. Deo Mishra (Bayer BioScience) highlighted work to develop BB resistant hybrids through MAB for commercial cultivation.

### TAL effectors: Role in infection

Genetic diversity among *Xoo* isolates includes variability in genes encoding a special class of virulence factors called Transcription Activator Like (TAL) effectors that are secreted into rice cells through the type III secretion system (T3SS). The role of TAL effectors in the process of infection of rice by both *Xoo* and another rice pathogen *Xanthomonas oryzae* pv. *oryzicola* (*Xoc*), was highlighted by Adam Bogdanove (Cornell University). His group cloned all the TAL effectors from a representative *Xoc* strain BLS256 and characterized those which serve as virulence factors in rice. The study established that host gene expression changes, especially those of TAL effector targets, are a valid phenotype for assessing strain diversity.

Valerie Verdier (Institut de Recherche pour le Développement, IRD) described the use of a novel strain of *Xoo*, X11-5A, which is devoid of TAL effectors, as a tool to evaluate TAL effector function one-by-one. A set of *Xoo* TAL effectors which activate known sugar transporter genes (i.e. *SWEET* genes) in rice were expressed in X11-5A as well as in *Xoc* and inoculated on different rice varieties. The results indicate that the *Xoo* TAL effectors which target rice *SWEET* genes contribute for virulence broadly and without tissue specificity. Boris Szurek (IRD) described identification of a novel source of broad spectrum resistance against *Xoo* from African germplasm; the resistance was identified based on impaired *SWEET14-* mediated susceptibility.

### Bacterial Virulence functions

Three broad themes emerged from this session on bacterial virulence mechanisms. These are detailed below:

### (I) *Xoo* cell-cell signaling

Subhadeep Chatterjee (Centre for DNA Fingerprinting and Diagnostics) provided an overview of the coordination of virulence traits in *Xanthomonas* by the production and perception of a Diffusible Signaling Molecule (DSF; a fatty acid like signaling compound). He highlighted the manner in which *Xoo* DSF controls transition from solitary (planktonic) to sessile (biofilm) lifestyle, a change that is crucial for pathogenesis. Chenyang He (Chinese Academy of Agricultural Sciences) described the identification of several novel and interesting *Xoo* proteins which coordinate quorum sensing-controlled virulence associated functions by affecting cyclic di-GMP synthesis and degradation. Vittorio Venturi (International Centre for Genetic Engineering and Biotechnology) described the role of a *Xoo* orphan receptor, OryR, which senses small molecular compounds derived from rice, and modulates regulation of virulence-associated traits. He further discussed how receptors such as OryR in many rice-associated bacteria may play a role in interspecies and inter-kingdom signaling.

### (II) Regulation of Type III secretion and control strategies

The bacterial T3SS is crucial for virulence of *Xoo*. Seiji Tsuge of Kyoto Prefectural University explained the role of HrpG and HrpX (master regulators) in the regulation of Xoo *hrp* genes (which encode the T3SS and its secreted proteins). He identified additional regulators involved in controlling *hrp* expression, in particular, the role of *Xoo* homologs of Histone-like nucleoid structuring protein (H-NS) (known as XrvB) and the Lon protease. Gong-You Chen from Shanghai Jiao Tong University provided a comprehensive picture of the complex regulatory patterns of the hrp regulon in *Xanthomonas* pathogens that infect rice. He further explained the role of HrcT, a component of the type III secretion in regulating the expression of HrpX regulon genes. Lin-Woo Kang (Konkuk University) presented structure-function studies of a cystathione β, ϒ-lyase (XometC) which is important for *Xoo* virulence and is a potential target for developing novel bactericides. Jeong-Gu Kim (National Academy of Agricultural Sciences in Suwon) described the development of a high-throughput screen to identify and quantitate biocontrol activity of several bacterial isolates and their active metabolites using a rice leaf and *Xoo* co-culture technique. Kim’s team has characterized two of these active compounds (staurosporin and naramycin), which have disease suppressing activities. These two compounds do not have a direct inhibitory role on *Xoo* growth and instead appear to work by boosting plant immunity through enhancement of the expression of plant stress/defense genes.

### (III) Induction and suppression of DAMP induced innate immunity

Ramesh V Sonti (CCMB) reported that several *Xoo* secreted proteins (such as cellulase, lipase, and xylanase) are involved in generating DAMPs (Damage associated Molecular Patterns) by hydrolyzing the rice cell wall. He indicated that four *Xoo* T3SS effectors, namely *Xanthomonas* Outer Protein N (XopN), XopQ, XopX and XopZ suppress rice innate immune responses that are induced by these DAMPs.

In the area of pathogen genomics, Prabhu Patil (Institute of Microbial Technology) explored the evolution of 34 Indian *Xoo* genomes. Phylogenetic reconstruction based on a set of constitutive genes shows high levels of diversification. The group of Ralf Koebnik at IRD has identified 16 loci suitable for Multi Locus VNTR Analysis (MLVA) in *Xoo* and *Xoc*. The VNTRs-based approach gives higher levels of resolution than other methods, and therefore emerges as a powerful tool for epidemiological surveillance of pathogen populations.

### The way forward

In the concluding session of the Conference, the following were discussed and agreed upon: (1) development and standardization of common genotyping and phenotyping systems and platforms, (2) build a well-curated bacterial germplasm collection comprised of isolates from different rice growing countries through strain exchange/transfer (3) engage in high-throughput pathogen monitoring and surveillance solutions, (4) study of changes in pathogen population structure in response to deployment of specific resistance genes, (5) establish and support a rice phytobiome partnership, (6) identification of suitable resistance gene/QTL combinations for durable BB resistance, (7) leveraging information gathered from analysis of virulence factors (such as TAL effectors) for effective resistance breeding and R gene deployment in rice, and (8) development of R gene containing NILs in the genetic background of Japonica rice.

The 5th ICBB will take place at IRRI in 2016.

